# Perfusion CT is a valuable diagnostic method for prostate cancer: a prospective study of 94 patients

**DOI:** 10.3332/ecancer.2014.476

**Published:** 2014-10-27

**Authors:** Elzbieta Luczynska, Pawel Blecharz, Sonia Dyczek, Andrzej Stelmach, Giuseppe Petralia, Massimo Bellomi, Barbara Alicja Jereczek–Fossa, Jerzy Jakubowicz

**Affiliations:** 1 Radiology Department, Centre of Oncology, M Sklodowska-Curie Memorial Institute, Cracow Branch, Cracow, Poland; 2 Gynecologic Oncology Department, Centre of Oncology, M Sklodowska-Curie Memorial Institute, Cracow Branch, Cracow, Poland; 3 Surgery Department, Centre of Oncology, M Sklodowska-Curie Memorial Institute, Cracow Branch, Cracow, Poland; 4 Radiotherapy Department, Centre of Oncology, M Sklodowska-Curie Memorial Institute, Cracow Branch, Cracow, Poland; 5 Radiotherapy Department, European Institute of Oncology, Milan, Italy; 6 Radiology Department, European Institute of Oncology, Milan, Italy; 7 University of Milan, Milan, Italy

**Keywords:** perfusion CT, prostate cancer, angiogenesis

## Abstract

**Purpose:**

The aim of this study is to assess the usefulness of perfusion computer tomography (pCT) in prostate cancer (PCa) diagnostics.

**Materials and Methods:**

94 patients with biopsy-proven PCa were enrolled in the study. Dynamic pCT of the prostate gland was performed for 50 seconds after an intravenous injection of contrast medium. Blood flow (BF), blood volume (BV), mean transit time (MTT) and permeability surface area product (PS) were computed in the suspected PCa area and in normal prostatic tissue.

**Results:**

PCa was visible in pCT in 90 of the 94 examined patients as a focal peripheral CT enhancement. When PCa was located in the peripheral zone (PZ), it was visible on perfusion maps, mostly showing an early peak followed by wash-out. The average values of all perfusion parameters were higher for tumour than for normal prostate tissue (*p* < 0.000). BV and BF were dependent on tumour grade expressed by the Gleason score (GS). All PCa cases were divided into groups, according to histological grade, as low (GS ≤ 6), medium (GS = 7), and high (GS > 7). In high-grade PCa, the mean BF value was significantly higher (*p* = 0.001) than the mean value of BF low- and medium-grade PCa (*p* = 0.011). Similar results were obtained regarding the mean values of BV; the more aggressive the cancer grade, the higher the mean BV value (*p* = 0.04).

**Conclusion:**

CT quantitative perfusion imaging allows PCa to be distinguished from normal prostate tissue. The highest values for BF and BV were observed in the most aggressive PCa grade.

## Introduction

In terms of frequency and incidence, prostate cancer (PCa) is the second most common malignant tumour in Europe and the United States [1]. Because of its biology, PCa has become a chronic disease, responding well to hormonal therapy; moreover PCa usually afflicts older patients with coexisting disease. This is why prognostic factors, like local spread of the tumour, influencing the choice of more aggressive treatment are still under debate [2].

The major goal in PCa imaging is to increase the tumour detection accuracy of and characterisation through the synthesis of anatomic, functional and molecular imaging. Standard diagnostic computed tomography has limitations in respect to both detection and staging of PCa. These limitations are due to the unclear distinction between the cancer and the adjacent normal tissues and benign prostatic hyperplasia [3]. Since the local progression of the tumour remains the crucial prognostic factor, CT is not indicated for the assessment of PCa staging because of its lack of efficiency in visualising foci within the gland, and poor visualisation of its zonal structure and anatomical capsule. The accuracy of CT is low in diagnosing neoplasm infiltration beyond the organ. Therefore, CT is required only when the serum PSA level is >20 ng/ml, the Gleason score (GS) is > 7 and the clinical tumour stage is T3 or higher [4, 5]. More recently, magnetic resonance imaging (MRI) is becoming the gold standard in the local tumour extension definition [6]. However, the group of patients with contraindications for MRI like claustrophobia, metallic endoprosthesis, pace-makers (non MRI-compatible), and stents must be considered; moreover, MRI devices are not widespread in lower-middle-income countries and prostate MRI interpretation requires experience in functional imaging techniques, such as diffusion-weighted MRI, dynamic contrast-enhanced MRI (DCE-MRI) and magnetic resonance spectroscopy (MRS). They make the PCT as a promising diagnostic tool in the selected groups of patients [7].

The assessment of perfusion characteristics is promising for PCa detection and characterisation. The rationale for using such imaging modalities is based on the observation that PCa has a different vascular pattern to that of normal tissue. It has been shown that the density of pathological vessels (microvessel density, MVD) within PCa was correlated with the presence of metastases, stage of disease, and survival rates, which can provide data potentially useful for therapeutic decisions and can be considered a surrogate biomarker of tumour angiogenesis [8, 9].

The usefulness of PCT was recently analysed in lung, cervical, head and neck, and other solid tumours [10–14]. Perfusion analysis of PCa has primarily been used in the fields of ultrasound and MRI [15–16]. Previous research have also demonstrated the usefulness of CT in PCa and proved the correlation between PCT parameters, histopathological findings, and MVD [17–19]. However, there is a lack of data on the usefulness of PCT in the diagnosis of PCa. Therefore, the aims of our study were to investigate the sensitivity of PCT for differentiating PCa from normal prostate tissue and to verify the differences in quantitative perfusion parameters between PCa and normal prostate tissue.

## Materials and methods

The study protocol was granted by the Polish Ministry of Science and Higher Education, research grant NN403240837 titled ‘CT perfusion and biological markers in local staging and risk assessment in surgically treated PCa patients’. The study was approved by the local Ethics Committee and prior written informed consent was obtained from all patients.

### Study design

In brief, the study design includes the prostate PCT, followed by prostate biopsy (performed two weeks after PCT). If prostate malignancy is positive, radical prostatectomy should be performed in the following two weeks.

Study procedures include:

1. Prospective registration of the eligible patients, signing of informed consent.
2. Execution of PCT.
3. Evaluation of PCT colour maps and computation of the following perfusion parameters: blood flow (BF), blood volume (BV), permeability-surface area (PS) and mean transit time (MTT).
4. Execution of core biopsy.
5. Radical prostatectomy.
6. Analysis of correlations between BF, BV, PS, MTT and histopathological findings.

## Patients

Between 2007 and 2011, a total of 94 patients with PCa median age 63 years; (range 49–77) met the selection criteria and were enrolled in the study: patients with a serum PSA level > 4ng/ml ranging from 4.4 to 39 ng/ml and mean value of 9.8 ng/ml. TRUS examination, abdominal and pelvic CT, and prostate gland perfusion. Prior to CT examination, the creatinine level was determined as being lower than the serum creatinine level ≤ 1 mg/dL [14]. The study was approved by the Local Ethics Committee and prior written informed consent was obtained from all patients. A Core biopsy of the prostate gland was performed on all patients. In the analysed group, there were 54 (60%) low grade tumours with GS ≤ 6, 31 (34%) medium grade tumours with GS 7 and 5 (6%) high grade tumours with GS > 7.

The patients were prospectively enrolled if they met the following criteria: elevated serum PSA level (>4 ng/ml), histologically proven PCa and neoplastic process confined to prostate gland confirmed in imaging examinations (TRUS, tru-cut biopsy and abdominal CT). Patients with previous neoplasms at other sites, not treatable or allergic to iodinated contrast media, were excluded from the study.

## Technical study

CT examinations were performed with a 16-section multidetector CT (MDCT) scanner (LightSpeed 16; GE Healthcare, Milwaukee, Wisconsin, USA). A preliminary, non-contrast CT of the pelvis (5-mm thickness) was performed to localise the prostate. The scanning range for the pCT was chosen to include two levels: upper (up to 2 cm above the centre of the prostate gland) and lower (2 cm below the centre), which together covered an area of 4 cm (top-bottom dimension). A total of 100 ml (2×50 ml) of non-ionic iodinated contrast material was injected (Ultravist 370 mg I/ml; Bayer Schering Pharma, Germany) followed by 50 ml of saline solution, at a rate of 5 ml/s, via an 18-gauge cannula placed in the right antecubital vein to exclude any source of variability. pCT scanning started 5 seconds after contrast administration with the following parameters: 4 contiguous 5-mm reconstructed sections at a constant table position, 1-second gantry rotation time, 120 kVp and 180 mA. Images were acquired every second for 50 seconds.

Immediately after completion of pCT scanning, a diagnostic CT of the abdomen and pelvis was performed using 5mm slices, standard reconstruction filter, 180 mA; 120 kV(p); rotation time, 0.6 seconds; speed, 9.38 mm/rotation; FOV 18 cm with intravenous contrast administration during perfusion scan (second retro-reckon 1.25 mm slice thickness).

The obtained images were transferred to an image-processing workstation (Advantage Windows 4.2, GE Healthcare) and analysed with commercially available software (CT Perfusion 4, GE Healthcare). The arterial input was obtained from a standardised region in the external iliac artery (EIA), with selection of the section that allowed best visualisation to avoid partial volume artefacts. A time-attenuation curve, expressed in Hounsfield units (HU) per second, was generated for the arterial input. Its geometric evaluation allowed readers to assess the timing of the pCT scans in each patient, excluding any early enhancement, identifying correctly the end of the first pass of contrast material and excluding any recirculation effect in the pCT measurements.

**Figure 1.**
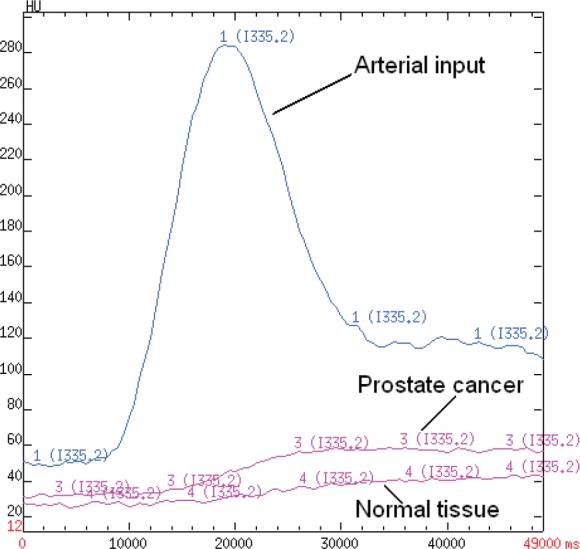
A time-attenuation curve for PCa and normal tissue. A density–time curve obtained by placing an ROI over the selected arterial input of external iliac artery, neoplastic tissue and healty prostate tissue plotting density (expressed in Hounsfield units (HU)) on the Y-axis and time (expressed in milliseconds) on the X-axis.

For qualitative analysis, dynamic CT images and perfusion maps were assessed. The analysis of dynamic CT images was performed with the cine-loop tool of perfusion CT 4.0 software. The focal areas showing the fastest and strongest contrast enhancement within normal enhancing prostate tissue were identified. An electronic cursor was then placed on those focal areas to visualise the corresponding density–time curve on the dedicated monitor partition. Obvious PCa was defined as a fast enhancing focus within normal enhancing prostate tissue showing fast initial contrast enhancement, followed by a plateau (change after maximum peak < 10% HU) or wash-out (decrease after maximum peak > 10% HU) in the delayed phase (Figure 1).

For quantitative analysis, a region of interest (ROI) was manually drawn along the visible margins of the obvious PCa by electronic cursor, in all the sections in which tumour was visible (area range: 48–221 mm^2^, mean value: 128 ± 46 mm^2^) and saved for each patient.

**Figure 2.**
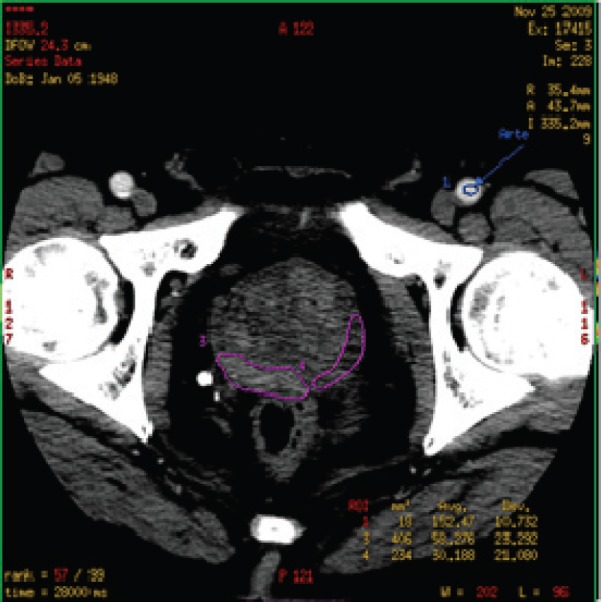
Region of interest (ROI) in prostate CT perfusion: the manually drawn ROI containing malignant tissue (purple contour on the right side of prostate gland) and healthy tissue (purple contour on the left side of prostate gland).

ROIs were chosen so that on all images they were drawn over regions of tumour throughout the image series irrespective of motion. If excessive motion artefacts precluded drawing a region of interest that stayed within the tumour margins in all the images of the CTp scan, the patient was excluded from the study (Figure 2).

Functional maps of blood flow (BF), blood volume (BV), and mean transit time (MTT) were generated according to the central volume principle that relates to BF, BV, and MTT by the equation: BF = BV/MTT [17].

**Figure 3.**
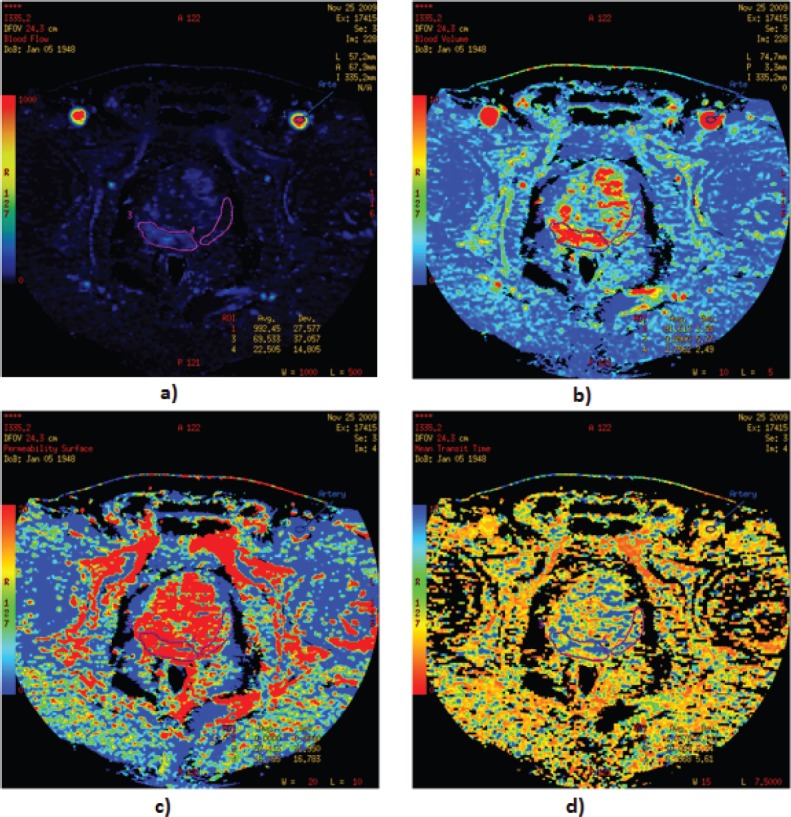
Functional maps of blood flow: (a) blood volume, (b) permeability surface, (c) mean transit time, (d) for PCa and normal tissue. Visualisation of functional maps of PCT parameters, with a focus on regions of interest is presented.

Tumour perfusion parameters were averaged over all sections of the ROIs drawn for each tumour. For display purposes, the functional maps were presented by using a colour scale with pixel values of BF measured in millilitres per 100 g wet tissue per minute; BV in millilitres per 100 g of wet tissue; MTT in seconds; and PS in millilitres per 100 g of wet tissue (Figure 3 a–d).

Colour maps were displayed in a rainbow scale (thresholds for BF = 0–400; for BV = 0–10; for MTT = 0–10, for PS = 0–20; the levels of these values were constant, to enable the reproducible colour scale on perfusion images), where higher values were shown in red and the lower ones in blue.

## Histopathology

Following radical prostatectomy, before obtaining the samples, the left and right side of the gland was marked to avoid confusion. The material was later sent to the Pathology Department in our Institute, where histopathological analyses were performed in the whole prostate tissue, by cutting the gland every 5 mm from apex to base to obtain the slides. Haematoxylin-eosin-stained specimens were examined microscopically in each case. Experienced pathologist, blinded to CT perfusion results, evaluated the whole set of slides to assess malignancy according to GS.

## Results

### Study population

**Figure 4.**
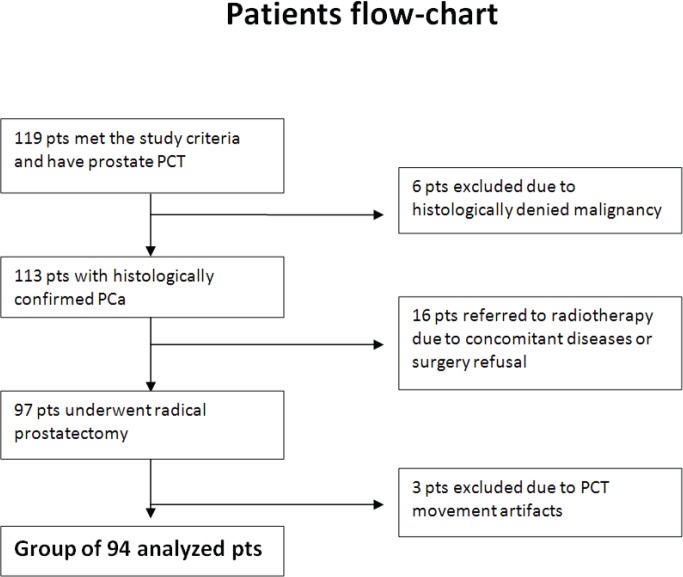
Flowchart of patients enrolled in the PCT study. The process of patients’ qualification to the study from included cohort to the final group subjected to analysis. Pts: patients.

Between 2007 and 2011, abdominal and pelvic CT and prostate gland perfusion CT were performed in 127 patients who were eligible for the study protocol and signed informed consent. A total of 94 patients with PCa (median age: 63 years; range: 49–77) met the selection criteria and were enrolled in the study 1 in the years 2007–2011 (Figure 4). The characteristics of the study population are shown in Table 1.

### PCT criteria

All patients demonstrated enhancement of periurethral tissue and of the hyperplastic transition zone. Ninety subjects were presented with focal enhancement in the peripheral zone of the prostate. In 34 (38%) patients, pathological foci were diagnosed on two levels, while in the remaining group only one focus was identified. Within this group, there were 13 (14%) patients with one tumour focus on the upper level and 43 (48%) patients with one tumour focus on the lower level. Despite the analysis of two foci, only one result (mean value of the perfusion parameters) was considered for each tumour.

Contrast enhancement typical of malignant lesions was visible in 90 of 94 patients (95.74% sensitivity for the method). Fast contrast enhancement appeared to be the dominant enhancement pattern for PCa (seen 17.4 ± 5 s (mean ± SD) from the start of contrast administration relative to the normal peripheral zone, i.e. 24.6 ± 9 s). Contrast enhancement appeared as a density-time curve characterised by an early peak in 24 (25.5%) patients. The remaining 70 patients had a density-time curve with a plateau in 22 (23.4%) or a continuous increase in 72 (76.65%). The characteristics of four patients with false negative PCT presents Table 2.

**Figure 5.**
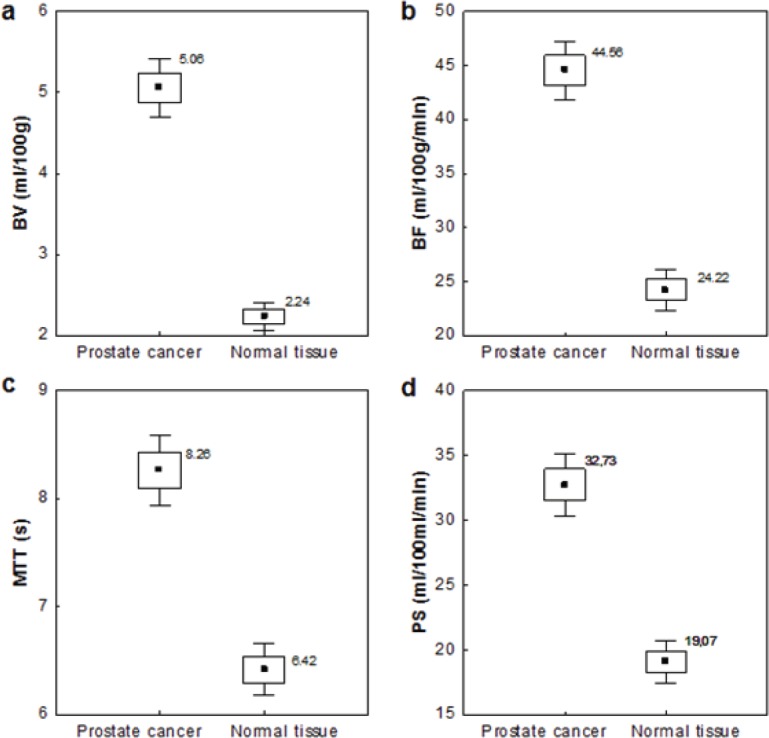
Differences in perfusion parameters between PCa and normal prostate tissue. The values of CT perfusion parameters. (a) blood volume, (b) blood flow, (c) mean transit time, (d) permeability-surface in malignant and healthy tissue. ■ Mean value, £ Mean ± SE, I Mean ± 1,96*SE.

Quantitative analysis was performed on the areas of tumour manually drawn throughout the image series (mean ROIs area value, 128 ± 46 mm^2^; range, 48–221 mm^2^) and on the areas of normal tissue (mean ROIs area 139 ± 51 mm^2^). The average values of the perfusion parameters that were analysed, namely BV, BF, PS, and MTT, were significantly higher (*p* = 0.000) for tumour than for normal prostate tissue (Figure 5).

## Discussion

The initial treatment of prostate cancer is generally based on clinical staging (digital rectal examination, prostate-specific antigen, biopsy assessment and imaging for metastatic disease), as well as the patient’s comorbidity. Transrectal ultrasonography (TRUS) allows the determination of the prostate size and depicts zonal anatomy, but its ability to delineate cancer foci is limited, with sensitivity and specificity of about 40–50%. Furthermore, the sensitivity of TRUS-guided tru-cut biopsy is only 39–65%, although can be improved with contrast enhancement [18].

Perfusion MRI and CT are based on an understanding of the process of tumour neoangiogenesis. With the development of new multi-detector computed tomography (MDCT) scanners, quantitative CT imaging of angiogenesis has become possible over large volumes of tissue. Although MRI is currently regarded as a gold standard in PCa imaging, the MDCT is also widely used as a general imaging method for evaluating cancer angiogenesis following the intravenous administration of exogenous contrast medium [19]. Using this technique, some authors have proved that perfusion parameters correlate with microvascular density or vascular area in differentiation of normal tissue from the tumour [19].

Although MRI is a method of great value in the work-up of PCa, some contraindications to this examination still exist, as well as limitations in access to the MRI devices in low-income countries [20]. Optional methods, like perfusion-computed tomography, could be a valuable alternative to TRUS and MRI.

Our study reveals significant differences between all perfusion parameters, i.e. blood flow (BF), blood volume (BV), mean transit time (MTT), permeability-surface (PS) area product, and normal prostatic tissue were found, what confirmed the usefulness of PCT in the detection of PCa in the prostate peripheral zone. The perfusion values obtained in tumours may reflect the process of angiogenesis. It has been suggested that high values of BF, BV, and PS measured using PCT are the result of the recruitment and development of arterio-venous shunts, dilated capillary beds, and hyperpermeable vessels.

To the best of our knowledge, our study is the first observation of sensitivity in PCT imaging of PCa. The number of our patients diagnosed with PCT is also the highest in the literature. The history of application of PCT in prostate tumour imaging is not long. Although the potential role of helical computed tomography in PCa was described in 2000 [21], the first paper on the role of perfusion CT in PCa was published by Ives *et al* in 2005 [22]. He found that a visible enhancement of PCa during dynamic CT is present only in half of the ten patients. In 2008, the identical method of PCT data analysis was used in 24 PCa patients treated with radical prostatectomy and revealed a very high correlation between perfusion parameters and histopathological findings [23]. Both authors used automatic ROI in the analysis, although some others suggest that manual method, used in our study, allows to delineate the malignant tissue more precisely [12, 24].

Recently, Osimani *et al* first used a 64-section MDCT scanner in PCT of PCa patients [19]. He confirmed that PCT parameters correlate well with microvessel density. He obtained the visualisation of malignant foci in 22 from 24 tumours, and demonstrated substantial differences in mean values of BV, MTT, and PS between PCa, BPH, chronic prostatitis, and healthy tissue. ROC curve showed 100% sensitivity and specificity for BV and MTT to discriminate benign and malignant lesions on prostate gland [19]. On the contrary, our study has the largest number of patients in the literature, which allows calculation of the sensitivity of PCT in the differentiation of PCa from normal prostate tissue. In our study, highly significant differences between mean values of BV, BF, MTT, and PS in normal tissue and PCa were demonstrated.

Perfusion rate was found to be an independent predictor of tumour response to chemo/radiotherapy and could be used to monitor recurrent tumours, for example, in squamous cell carcinoma in the upper aerodigestive tract or metastatic colorectal cancer the [25–27]. Altered perfusion parameters can be observed also in the pancreatic tumours and in the cervical lymph nodes of patients with squamous cell cancer of the hypopharynx and larynx [28, 29]. In our study, values of all perfusion CT parameters allowed to discriminate neoplastic from healthy prostatic tissue. Further investigation should focus on the comparison of the MRI and PCT in the same group of patients in order to define better the PCT advantages and limitations.

One of the potential areas of PCT employment in the PCa management might be radiotherapy treatment planning. Radiotherapy is one of the basic treatment options in PCa offering excellent tumour control with acceptable toxicity [30]. There is a high level evidence of the improved tumour control with the dose escalation [31, 32]. Standard CT is routinely used for planning, and commonly the whole prostatic gland is delineated as a clinical target volume (CTV). Therefore, further investigation in this area might be helpful to evaluate usefulness of the PCT in the identification of the dominant lesions allowing for a functional approach to the radiotherapy planning. Some authors suggest that MRI and PET-based perfusion targets reflect gross tumour volume (GTV) better than static imaging [33–35]. Our results imply that PCT can also be a valuable method of functional radiotherapy planning in the selected group of patients. Importantly, PCT-based planning would be a more cost-effective procedure when compared to MRI and PET-CT, especially in lower-middle-income countries.

Our study has some limitations. One is the lack of a control group, which did not allow calculation of the specificity of the method. Another one is the poor visibility of the anatomy of the prostatic capsule. PCT does not allow the accurate definition of the borders of cancer in the peripheral zone or its infiltration to other prostate anatomical areas or its capsule. This limitation is of great importance, especially in the case of the patient candidates for nerve-sparing surgery, when the tumour is ideally confined to the prostate gland [36]. Data analysis revealed higher perfusion parameters among patients with more advanced disease estimated in the Gleason score. Patients with a higher degree of malignancy in the Gleason score are also more advanced clinically, which resulted in a worse post-operative prognosis.

Another important limitation is looking for pathological focus of enhancement only in the peripheral zone, which could result in discounting cancers developing in the central or transitional zone.

A further limitation of the PCT method is the additional exposure of patients to radiation. In our study, the mean radiation dose in 2 cm examination was equal to 16.15 mSv. This dose is higher than that from a conventional CT scan of the pelvis, but still acceptable for whole body examination, particularly for patients where MRI is not suitable or available.

Because PCT is usually performed together with a conventional diagnostic CT of the examined region, the actual radiation dose is higher than the applied for the oncologic patient. It was necessary then to obtain an approval of the institutional ethics committee and written informed consent signed by all participants before the study. Unfortunately, such a high exposure dose makes it difficult to find a large number of homogeneous patients and draw valid scientific conclusions.

The lack of ability to visualise the prostate tumour before perfusion imaging is another inconvenience. It was therefore not possible to place the 20 mm volume of perfusion scans over the tumour region in all patients. The volume to be studied is limited along the z-axis by the number of detectors of the CT scanner, because the scans with such a high temporal resolution are only possible with a stationary table position.

During the examination, the volume of 4 cm was scanned. The diameter of a normal prostate gland was about 4 cm, but every year the gland enlarged from about 0.2 to 1.2 g. Sometimes the diameter of prostate gland reached 5 or 6 cm. It could be the reason why in many cases, our scanning was unable to visualise the entire organ.

## Conclusions

In conclusion, our study has demonstrated the high sensitivity of PCT for differentiation of PCa from normal prostate tissue. The values of perfusion parameters are significantly higher in tumours than in normal tissue, which may be helpful for the improved detection of malignancy. Our results might have an impact on daily practice, particularly in clinical situations where routine access to MRI for PCa is not feasible.

## Conflict of interest

The authors declare that they have no conflict of interest.

## Figures and Tables

**Table 1.** Baseline characteristics of 94 patients diagnosed with PCT.

Characteristics	*n* = 94	%
**Age (years)**		
≤65 years	57	60.6
65–70 years	23	24.5
70–75 years	13	13.8
>75	1	1.1
**pT stage**		
pT2	60	63.8
pT3	34	36.2
**pN stage**		
pN0	90	95.7
pN1	4	4.3
**Gleason score (surgical)**		
≤5	25	26.6
6	42	44.7
7	24	25.5
8–10	3	3.2
**Histological grade**		
Low (Gleason ≤ 6)	67	71.3
Moderate (Gleason = 7)	24	25.5
High (Gleason ≥ 9)	3	3.2
**Initial PSA (ng/ml)**		
4–10	61	64.9
10–20	29	30.8
>20	4	4.3
**Initial NCCN risk of recurrence groups** [21]		
Low	1	1.1
Intermediate	87	92.5
High	6	6.4
**Total**	94	100

Abbreviations: pT, pN: pathological TNM classification;

NCCN – National Comprehensive Cancer Network

**Table 2.** Characteristics of four PCa patients with false negative PCT.

Patient	Age	PSA level (ng/ml)	Gleason score	pTNM stage	NCCN risk of recurrence group
1	63	5.5	7	T2N0M0	Intermediate
2	67	6.9	6	T2N0M0	Low
3	66	4.0	6	T2N0M0	Low
4	55	18.5	5	T2N0M0	Intermediate
